# Pain-related function, muscle stiffness, and muscle activation in adults with patellofemoral pain

**DOI:** 10.1186/s12891-026-09710-3

**Published:** 2026-03-18

**Authors:** Ge Zhu, Yuqing Jia, Yuang Hao, Xin Miao, Enming Zhang

**Affiliations:** 1 Department of Sports Medicine, Peking University Third Hospital, Beijing, 100191 China; 2 School of Sports Medicine and Rehabilitation, Beijing Sport University, Beijing, 100084 China

**Keywords:** Patellofemoral pain, Anterior knee pain, Shear wave elastography, Electromyography

## Abstract

**Background:**

Patellofemoral pain (PFP) is a prevalent knee condition affecting adolescents and active adults, characterized by diffuse anterior knee pain exacerbated by weight-bearing activities and knee flexion. The onset and improvement of PFP are believed to be related to the condition of the quadriceps muscles. However, there remains a lack of rapid, convenient, and accurate clinical tools for assessing the characteristics of muscles. Our research aimed to explore muscle stiffness in three states (resting, stretching, and contraction), as well as to evaluate muscle activation, and pain-related function in adults with patellofemoral pain (PFP). And to assess the diagnostic value of shear wave elastography (SWE).

**Methods:**

Eighteen subjects with unilateral PFP (39.8 ± 11.9 years) and 20 control subjects (30.3 ± 8.2 years) underwent muscle stiffness measurements in resting, stretching, and contraction positions. Quadriceps muscle stiffness and surface electromyography (sEMG) data were collected. VMO/VL activation and stiffness ratios were analyzed between groups. Self-report questionnaires (AKPS and KOOS-PF) assessed pain and knee function.

**Results:**

At resting, no significant differences were found in the stiffness of the VMO, VL, or RF between groups. During stretching, VMO stiffness was significantly lower in PFP limbs compared to pain-free controls (*p* = 0.032), while VL stiffness was significantly higher in PFP limbs compared to both asymptomatic limbs (*p* = 0.019) and controls (*p* = 0.008). During contraction, VMO stiffness was significantly lower in PFP limbs compared to asymptomatic limbs (*p* = 0.039). The VMO/VL ratio was significantly reduced across three conditions. Combining VMO/VL ratios during stretching and contraction yielded the highest diagnostic performance, with an area under the curve (AUC) of 0.832, specificity of 80%, and sensitivity of 66.67%.

**Conclusion:**

Individuals with PFP exhibited significant impairments in the VMO/VL stiffness ratio. Specifically, the VMO stiffness decreased during contraction, while VL stiffness increased during stretching. Integrating VMO/VL stiffness from stretching and contraction positions enhanced diagnostic accuracy for PFP.

**Supplementary Information:**

The online version contains supplementary material available at 10.1186/s12891-026-09710-3.

## Introduction

Patellofemoral pain (PFP) is a prevalent knee condition affecting adolescents and active adults, characterized by diffuse anterior knee pain exacerbated by weight-bearing activities and knee flexion. The squat test is a reliable method for diagnosing PFP [1]. Reports indicate that the prevalence of PFP is 22.7% in the general population and 28.9% in adolescents [2, 3]. Annual incidence ranges from 4% to 21% among leisure runners [4–6], and reaches 35.7% among male cyclists—with 6.4% of these symptomatic cyclists enduring symptoms for more than 30 days [7]. Patellofemoral joint disorder can lead to cartilage damage, chondromalacia, and cartilage delamination, which may ultimately progress to knee osteoarthritis [8, 9]. This condition and its progression can impact patients’ physiological, psychological, and social functioning [10, 11].

However, the pathogenesis of PFP is considered multifactorial and remains largely unknown. Powers et al. [12] linked it to abnormal patellofemoral joint (PFJ) loads [13]. Key factors include PFJ contact area, joint alignment, knee/adjacent joint dynamics, and muscle properties [12]. Notably, abnormal quadriceps tension—encompassing active and passive components—may play a pivotal role: disproportionate VMO-VL activation (active tension imbalance) disrupts patellar tracking, while elevated passive tension (e.g., fascial adhesions) restricts mobility and amplifies compressive forces. Despite extensive exploration of muscle morphology (e.g., thickness) and basic activation patterns [13, 14], few studies have comprehensively evaluated muscle tension—specifically, the combined effects of active tension and passive tension on PFP symptoms.

In addition, current methods for quantifying muscle status each have limitations. Joint range of motion (ROM) measurements only indirectly reflect muscle flexibility [15] and cannot quantify internal muscle mechanical properties; surface electromyography (sEMG) describes muscle function via electrical activity but involves complex operation and signal processing, leading to unstable results [16]. MRI and ultrasound (US) offer high spatial resolution for tracking muscle morphological changes but have low temporal resolution; additionally, MRI is costly and time-consuming, limiting its routine clinical use [17, 18]. Advanced techniques like finite element analysis (FEA) [19], discrete element analysis [20], and artificial intelligence (AI) [19] simulate muscle deformation and stress distribution under external forces but rely on extensive real-world data for validation, restricting their clinical translation. Regarding the treatment of PFP, an amount of clinical evidence [21] and guidelines [22] have underscored the importance of muscle training for the knee and hip joints. Therefore, it is imperative to use appropriate tools to assess muscle characteristics in patients with PFP.

In contrast, shear wave elastography (SWE), as an innovative ultrasound technique, can acquire shear modulus values by emitting and tracking shear waves, thereby realizing real-time and dynamic quantification of local muscle stiffness [23, 24], representing muscle contraction ability [25–27]. It also has the advantages of simple operation, non-invasiveness, and repeatability [28, 29]. Previous studies have confirmed that SWE measurement results are correlated with muscle strength [30] and functional tests [31] in healthy populations, suggesting its potential in reflecting muscle structural and functional status. SWE quantifies stiffness as a surrogate for passive tension (at rest and stretching) and active tension-related changes (during contraction), while concurrent sEMG during contraction assesses neuromuscular activation patterns to complement active tension analysis.

Therefore, this study focused on muscle stiffness as the core biomechanical evaluation parameter and employed Shear Wave Elastography (SWE) to quantitatively assess the target muscles in patients with patellofemoral pain (PFP) under three distinct functional conditions. Simultaneously, surface electromyography (sEMG) was applied synchronously to capture the electrophysiological activity of the same muscle groups during contraction, providing supplementary information on muscle functional status.

## Method

### Study design

This study was conducted from April to November 2024, adhering to the Declaration of Helsinki and following the STROBE guidelines [32]. All procedures were approved by Peking University Third Hospital Medical Science Research Ethics Committee (IRB00006761-M2023425). Written informed consent was obtained from all participants, ensuring their rights were protected.

### Recruitment

Posters were placed in physical therapy and medical clinics to recruit individuals with unilateral PFP and healthy controls. A power analysis using G*Power 3.1.9 (Heinrich-Heine-Universität, Düsseldorf, Germany) indicated that 38 participants (19 PFP patients and 19 controls) were needed for a power of 0.90, an effect size of 0.7, and an α = 0.05.

### Participants and diagnostics

The clinical examination was conducted by one of two physical therapists with 4 and 7 years of experience, respectively. PFP diagnosis was based on established criteria [1, 33]—insidious onset of anterior or retropatellar knee pain for more than 6 weeks; pain provoked by at least two activities (prolonged sitting/kneeling, squatting, running, hopping, stair climbing); tenderness on patella palpation; or pain during stepping down or double-leg squatting. Participants also needed to report ≥ 30 mm on a 0-to-100-mm visual analog scale for worst pain in the past week. Exclusion criteria included any history of lower extremity surgery or significant injury leading to non-weight bearing, back pain, internal knee derangement, or other sources of anterior knee pain (patellar tendinopathy, Hoffa fat-pad syndrome, infrapatellar/suprapatellar bursitis, patellar dislocation/subluxation). Participants with pain isolated to the inferior pole of the patella were also excluded.

### Data collection

#### Pain-related function

To assess pain-related function, the respective subscales from the Chinese-translated versions of the Anterior Knee Pain Scale (AKPS) [34] and the Knee Injury and Osteoarthritis Outcome Score - Physical Function Short Form (KOOS-PF) [35] were used. The AKPS and KOOS-PF questionnaire are widely used and well-validated questionnaire for assessing the severity of symptoms and physical limitations in people with PFP [21, 33, 36, 37].

#### Muscle stiffness

To evaluate muscle elastic modulus in different states, this study used a portable dynamic ultrasound elastography device (T5C1A304WT; Beijing Xijian Technology). The device includes two handheld probes: one for mechanical wave excitation and the other for shear wave capture. It applies 100 Hz resonant vibrations to induce shear waves within muscles, while B-mode imaging captures anatomical images and shear wave speed to determine tissue shear modulus. An 8.5 MHz probe with a footprint size of 32 mm × 4.4 mm was used for real-time visualization and data storage. A 2 mm gap filled with ultrasound gel was maintained between the probe and skin. All ultrasound measurements were performed by two experienced musculoskeletal ultrasound physicians hired as independent operators to ensure technical accuracy and measurement objectivity.

Participants were positioned supine on an adjustable bed and instructed to remain relaxed. Muscle identification used standard anatomical landmarks: rectus femoris (RF) at the midpoint between ASIS and patella’s superior border [38]; vastus lateralis (VL) two-thirds along the line from ASIS to the lateral edge of the patella [39]; vastus medialis oblique (VMO) at 4 cm above and 3 cm medially from the superior border of the patella [40, 41].

Shear modulus measurements were first taken from the anterior thigh muscles (including rectus femoris, vastus medialis oblique, and vastus lateralis) in a resting position with the participant lying supine and fully relaxed on an examination table. For the stretch condition, participants were repositioned with their lower legs hanging freely off the edge of the treatment bed, with knees flexed at 90° to ensure a stable stretch. Finally, for the contracted position, participants performed single-leg weight-bearing knee extension tasks from a 15° knee flexion position while supine, with a yoga block placed under the popliteal fossa. During this phase, two assessors simultaneously recorded data from the vastus medialis obliquus and vastus lateralis, with each assigned to one muscle. The rectus femoris was measured afterward using the same condition. Each condition was tested for 10 s with two repeated measurements. Verbal feedback ensured consistent pacing and smooth execution, and patients were instructed to keep toes pointing vertically toward the ceiling, extend their knees slowly with minimal force in the contracted position, and practice this pattern 3–5 times to prevent unintended rotation.

#### Muscle activation

**Fig. 1:**
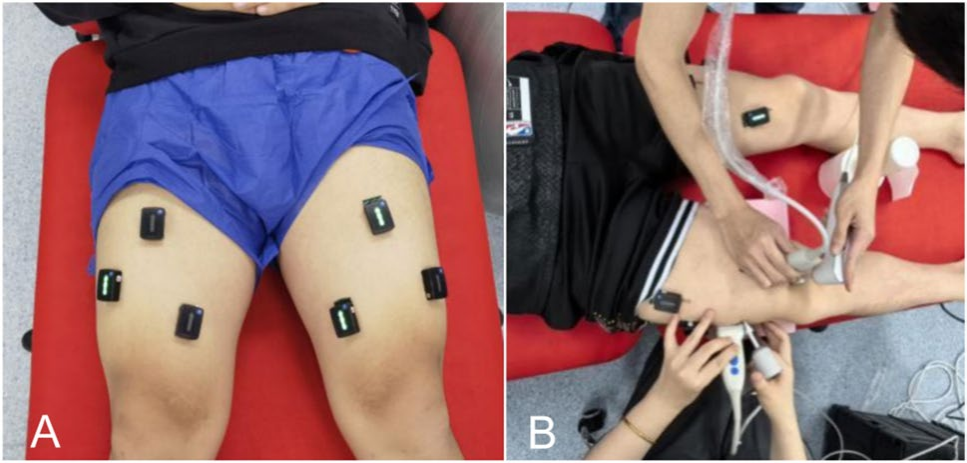
**A**: The placement of the EMG electrodes on the quadriceps; **B**:The methodology of shear wave elastography testing

Quadriceps femoris muscle activity was recorded using wireless surface EMG system (Trigno Sensor System, Delsys Inc., Natick, MA, USA: interelectrode distance = 10 mm, 80 dB common mode rejection rate) for RF, VL, and VMO on both sides of participants. EMG data were sampled at 2000 Hz. Figure 1 showed the placement of the EMG electrodes on the quadriceps and the methodology of shear wave elastography testing. Each subject lies supine on a height-adjustable treatment table, where surface electromyography (sEMG) electrodes are standardized according to muscle anatomy and the degree of muscle fiber prominence [40]. Before affixing the electrodes, the skin was cleaned with alcohol to ensure adequate contact. The EMG data were collected simultaneously with SWE (Shear Wave Elastography) during the patient’s active contraction, recording surface electromyography signals from three channels unilaterally [42]. And both techniques used concurrently with probe spacing maintained to prevent coupling gel from contaminating the electrodes.

### Statistical analysis

Quadriceps femoris muscle activity was recorded using Delsys surface electrodes for RF, VL, and VMO on both sides of participants. Prior to testing, maximal voluntary isometric contractions (MVIC) were obtained for normalization. Muscle activity onset and cessation were identified when the EMG signal exceeded or fell below 2 standard deviations of baseline for at least 20 ms. EMG onsets were verified visually to avoid misidentification. Raw data were transferred to MATLAB (MathWorks R2021a). EMG signals were band-pass filtered (10–300 Hz), low-pass filtered (6 Hz cutoff), and smoothed using RMS values with a 10 ms moving window. Peak data were normalized to %MVIC. A mean vastus medialis oblique/vastus lateralis (VMO/VL) muscle ratio was calculated for each subject.

For two repeated measurements per condition, the average of the two valid runs was used for analysis. The data were analyzed based on the study’s sub-problems. To compare the outcome between PFP patients and healthy individuals, all data were statistically analyzed using SPSS(26, SPSS Inc., Chicago, IL, USA). Paired sample t-tests and Wilcoxon Signed Rank Tests compared the affected and healthy sides in patients, while Mann-Whitney tests compared patients with healthy subjects. Paired t-tests assessed variable correlations, and logistic regression calculated joint disease probability for the diagnostic model. ROC curve analysis evaluated model accuracy, with AUC values closer to 1 indicating superior discriminatory power.

## Results

### Demographics

A total of 38 participants were recruited, comprising 18 individuals diagnosed with unilateral PFP and 20 healthy controls. Eleven (61%) of the 18 participants with unilateral PFP had symptoms in their dominant (kicking) limb. The demographics was showed in Table 1.

**Table 1:** Demographics

	PFP (*n* = 18)	Pain-Free Controls(*n* = 20)	*p*-value
Age, y	39.8 ± 11.9	30.3 ± 8.2	0.007
Weight, kg	83.7,25.3	73.6 ± 10.6	0.161
Height, cm	176.4 ± 4.9	173.7 ± 6.5	0.108
Body mass index(BMI), kg/m^2^	26.8 ± 7.7	24.3 ± 3.0	0.184
KOOS pain (0-100)^a^	62.8 ± 18.5	97.6 ± 5.2	<0.001
AKPS (0-100)^a^	81.4 ± 10.8	99.6 ± 1.7	<0.001

*Abbreviations*: *KOOS* Knee injury and Osteoarthritis Outcome Score, *AKPS* Anterior Knee Pain Scale, *PFP* Patellofemoral pain

^a^Higher score reflects greater function and fewer symptoms

### Muscle stiffness (SWE)

**Fig. 2:**
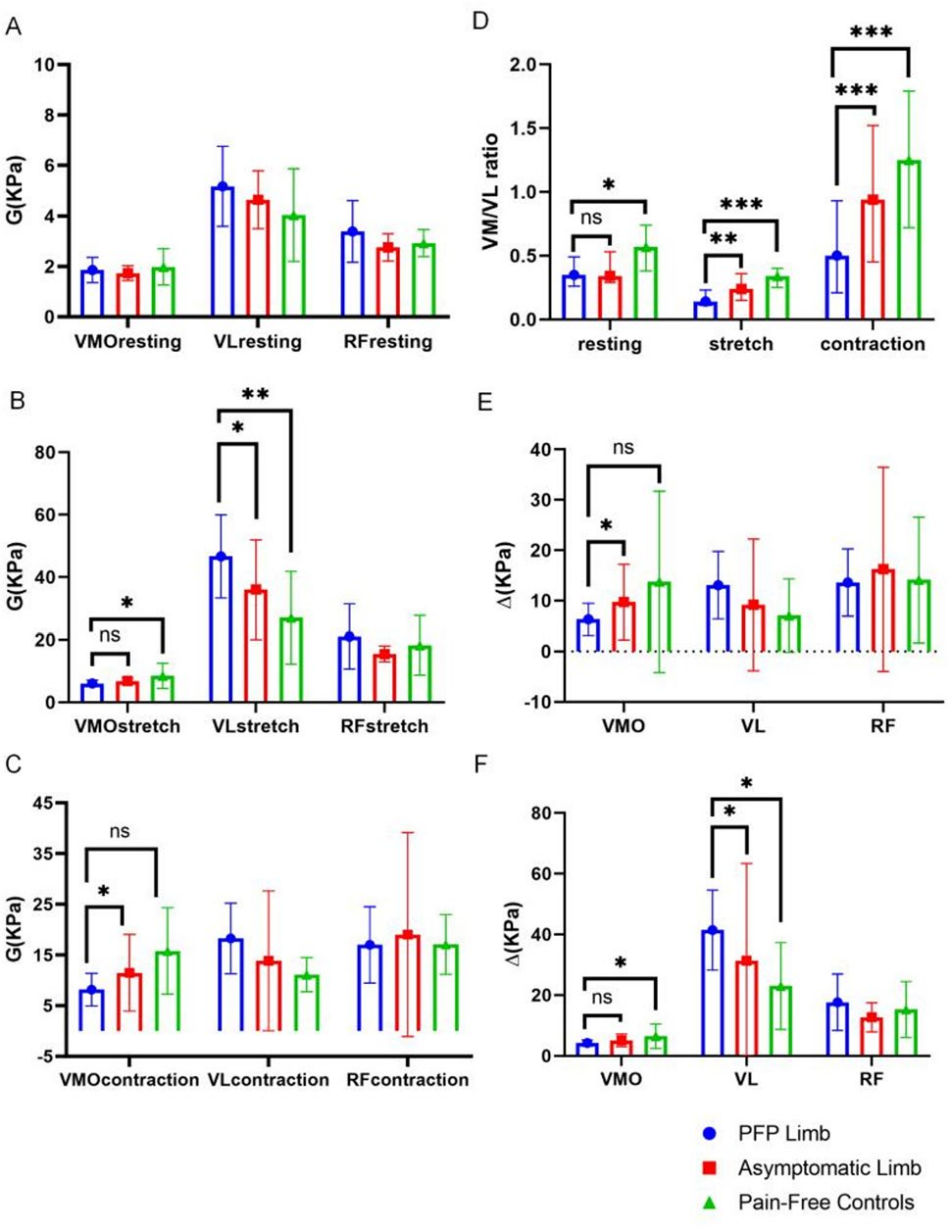
Comparison of SWE between symptomatic, asymptomatic limbs with unilateral PFP and Pain-Free Controls in resting **A**, stretch **B**, and contraction (**C**) position. **D**: Comparison of VMO/VL ratio between symptomatic, asymptomatic limbs with unilateral PFP and pain-free controls in resting, stretch, and contraction. **E**: Comparison of the difference between the contraction position and the resting position. **F**: Comparison of the difference between the stretched position and the resting position. Values are mean and 95% confidence interval. *Statistically significant difference, *p* < 0.05. **p < 0.01. ***p < 0.001. Abbreviations: PFP, patellofemoral pain; RF, rectus femoris; VL, vastus lateralis; VMO, vastus medialis oblique

Our study found the differences in muscle stiffness (Fig. 2), assessed using shear wave elastography (SWE), across symptomatic, asymptomatic, and pain-free control limbs in individuals with patellofemoral pain (PFP). The stiffness of the VMO during both stretch and contraction was significantly lower in the PFP limbs compared to pain-free controls (*p* = 0.032) and the asymptomatic limbs (*p* = 0.039).

Conversely, the VL stiffness during stretch was significantly higher in the PFP limbs compared to both asymptomatic limbs (*p* = 0.019) and pain-free controls (*p* = 0.008). The VMO/VL ratio was significantly reduced in the PFP limbs across multiple conditions, including in the resting state compared to pain-free controls (*p* = 0.011), during stretch compared to both asymptomatic limbs (*p* = 0.007) and controls (*p* < 0.001), and during contraction compared to both asymptomatic limbs (*p* = 0.008) and controls (*p* = 0.004). Additionally, the change in VMO stiffness between stretch and resting (*p* = 0.035) and contraction and resting (*p* = 0.043) was significantly lower in the PFP limbs compared to both controls and asymptomatic limbs, respectively. Furthermore, the change in VL stiffness between stretch and resting was significantly greater in the PFP limbs compared to asymptomatic limbs (*p* = 0.018) and controls (*p* = 0.011). There are more detailed result data in the supplementary file. These findings highlight alterations in quadriceps muscle properties and imbalances associated with patellofemoral pain (PFP).

### Muscle activation

Significantly greater muscle activation was observed in the VMO of the symptomatic limbs compared to the asymptomatic limbs in individuals with unilateral patellofemoral pain (*p* = 0.004). No significant differences in muscle activation were found between limbs for the VL and RF. But the VMO/VL activation ratio was significantly higher in the symptomatic limb than in the asymptomatic limb (*p* = 0.01). When comparing the muscle activation of the VMO, VL, and RF in the limbs of individuals with PFP to the matched limbs of control group participants, no significant differences were observed, including for the VMO to VL activation ratio (Table 2).

**Table 2:** Comparison of RMS Between Symptomatic, Asymptomatic Limbs with Unilateral PFP and Pain-Free Controls

	PFP limbs(*n* = 18)	Asymptomatic Limbs(*n* = 18)	Pain-Free Controls(*n* = 20)
VMO,%MVIC	22.35 ± 12.34	13.13 ± 7.02^*^	17.22 ± 12.60
VL, %MVIC	25.03 ± 11.45	26.58 ± 14.98	24.21 ± 14.29
RF, %MVIC	12.83 ± 6.65	13.77 ± 1.34	13.71 ± 7.77
VMO/VL	0.93 ± 0.23	0.55 ± 0.18^*^	0.76 ± 0.24

*Abbreviations*: *PFP* Patellofemoral pain, *RF* Rectus femoris, *VL* Vastus lateralis, *VMO* Vastus medialis oblique

^*^Significantly different from PFP limbs group

### Correlation between muscle stiffness, muscle activation and clinical symptoms

We calculated the correlation coefficients between muscle stiffness, muscle activation, and clinical symptoms, as measured using the Anterior Knee Pain Scale (AKPS) and the Knee Injury and Osteoarthritis Outcome Score - Physical Function Short Form (KOOS-PF). The stiffness of the vastus lateralis (VL) during stretch showed a significant negative correlation with AKPS (*r* = -0.642, *p* = 0.004), indicating that greater muscle stiffness in this condition was associated with worse knee function and more symptoms. In contrast, the root mean square (RMS) values of VL activation were significantly actively correlated with AKPS (*r* = 0.490, *p* = 0.039), suggesting that higher muscle activation in the VL was associated with better knee function. No significant correlations were found for other parameters, such as muscle states in the resting or contracted conditions, or for KOOS-PF scores across most measures. The table of results can be found in the Supplementary file. These results suggest that specific shear wave elastography (SWE) parameters, particularly VL stretch stiffness, could serve as valuable indicators for assessing the functional and symptomatic status of individuals with patellofemoral pain.

### The accuracy of combination with muscle stiffness and muscle activation for diagnosing pfp

We also explored the diagnostic accuracy of muscle stiffness parameters, measured using shear wave elastography (SWE), for identifying patellofemoral pain (PFP). The VMO/VL ratio during stretch demonstrated an area under the curve (AUC) of 0.762, with a cut-off value of 0.29, high specificity (95%) and moderate sensitivity (66.67%). Similarly, the VMO/VL ratio during contraction showed an AUC of 0.766, with a cut-off value of 0.34, specificity of 90% and sensitivity of 72.22%. The combination of the VMO/VL ratios during both stretch and contraction yielded the highest diagnostic performance, with an AUC of 0.832, specificity of 80%, and sensitivity of 66.67%. More detailed results can be found in the Supplementary file. These results suggest that while individual muscle stiffness parameters offer valuable diagnostic insights, combining these measures enhances the accuracy and reliability of diagnosing PFP.

## Discussion

This study assessed quadriceps stiffness in PFP patients using SWE across three states (rest, stretch, contraction). Our key findings are: (1) VL stiffness in PFP patients was significantly higher during stretch compared to their asymptomatic limbs and controls; (2) PFP patients had lower VMO/VL stiffness ratios (< 1) during stretch and contraction; (3) The VMO/VL activation ratio was higher in PFP limbs but remained < 1 across groups; (4) Combining SWE measurements from stretch and contraction positions improved PFP diagnostic accuracy (AUC = 0.832). The novelty of this study lies in highlighting the clinical relevance of stretch-position stiffness assessment—previously overlooked in PFP research—and integrating SWE (mechanical properties) and sEMG (electrophysiological activation) to evaluate quadriceps dysfunction comprehensively.

A key result of this study is that VL stiffness in PFP patients was significantly elevated in the stretch position compared to controls. Previous research [43] using SWE and MRI has indicated that increased stiffness in the VL is associated with greater lateral displacement of the patella. This lateral displacement can lead to heightened stress on the patellofemoral joint, potentially exacerbating pain in patients with PFP. Our finding aligns with this mechanism, further underscoring that VL stiffness (especially in the stretch position) plays a critical role in disrupting patellar tracking and driving PFP pathogenesis. Notably, this result also highlights the value of assessing stretch-position muscle stiffness: prior PFP studies rarely focused on this state, yet our data suggest it may be a more sensitive indicator of quadriceps dysfunction than resting or contraction positions.

Another critical result is that during stretch and contraction positions, PFP patients had a significantly lower VMO/VL stiffness ratio (consistently < 1) than their asymptomatic contralateral limb and healthy controls. Based on previous research [44], it has been demonstrated that the elastic properties of muscle are highly correlated with the level of muscle activity. This finding aligns with prior studies that identified VMO weakness as a key contributor to PFP [44], and further confirms that VMO-VL imbalance (manifested as low stiffness ratio) is a pathological feature of PFP. Clinically, this result has implications for rehabilitation [45, 46]: since the imbalance is more prominent in stretch and contraction positions, physical therapists should prioritize interventions targeting VMO activation and VL stiffness reduction (e.g., isolated VMO training, myofascial release for VL) to restore quadriceps balance.

In terms of muscle activation, our study found that the VMO/VL activation ratio in PFP patients’ affected limbs was significantly higher than in their asymptomatic contralateral limb and healthy controls. This result is inconsistent with previous research, which is likely influenced by the specific testing maneuver utilized—a low-load terminal knee extension conducted in a supine position. We hypothesize that during this low-demand task, PFP patients adopt an adaptive motor control strategy—they enhance VMO fiber activation to compensate for VL stiffness and stabilize the patellofemoral joint, which explains the elevated VMO/VL ratio. A secondary observation from this result is that the VMO/VL activation ratio remained < 1 across all groups (PFP and controls), suggesting that a low VMO/VL activation ratio alone may not fully account for PFP symptomatology; instead, the combination of activation imbalance and stiffness imbalance (as observed in earlier results) is more likely to drive pain.

A fourth key result is that combining SWE-measured VMO/VL stiffness across stretch and contraction positions enhances PFP diagnostic accuracy, with an AUC of 0.832. This improvement in performance underscores two critical attributes of SWE. First, multi-position SWE measurements capture more comprehensive information on muscle mechanical properties than single-position assessments, as they reflect tissue behavior across distinct functional states relevant to PFP. Second, SWE-derived stiffness directly targets mechanical properties closely linked to PFP pathogenesis, a feature that distinguishes it from traditional assessment tools like EMG. EMG has well-documented limitations in this context, including inconsistent results [47] and an inability to directly reflect muscle force [48]. By addressing these limitations, SWE emerges as a more robust and reliable tool for quantifying VMO/VL imbalance and supporting PFP diagnosis.

Beyond its diagnostic utility, SWE-measured muscle stiffness also demonstrates relevance to PFP symptom severity. Our data indicate that SWE-derived stiffness indirectly reflects symptom changes in PFP patients, as it correlates not only with quadriceps dysfunction but also with patient-reported symptom severity. This dual relevance—linking pathological markers to clinical symptoms—enhances SWE’s value in clinical practice. Specifically, serial SWE assessments can be used to monitor both symptom progression and treatment response over time, as changes in stiffness metrics can be interpreted alongside changes in patient symptoms. This application bridges the gap between objective biomechanical assessment and subjective clinical outcomes, providing a non-invasive, practical tool that supports both diagnostic decision-making and therapeutic monitoring in PFP management.

### Clinical implications

This study found that individuals with Patellofemoral Pain (PFP) exhibit impaired VMO to VL stiffness ratios, particularly during contraction. PFP patients showed significantly higher VL stiffness compared to asymptomatic limbs and controls during stretching. These findings substantiated the importance of incorporating targeted interventions aimed at enhancing VMO strength during contraction and reducing VL tightness within clinical rehabilitation protocols for PFP patients. Such strategies can help improve muscle balance and alleviate symptoms.

### Limitations

The present study was limited to assessing a supine low-load terminal knee extension movement. Subsequent investigations will expand to include a wider array of clinically pertinent motions to further elucidate the findings. As this is a cross-sectional study, suggestions based on the results of this study are speculative. And we will conduct clinical trials to further validate and investigate the clinical significance of muscle stiffness measurements obtained through shear wave elastography.

## Conclusion

The present investigation revealed that individuals with Patellofemoral Pain (PFP) demonstrate substantial impairments in the stiffness ratio between the Vastus Medialis Oblique (VMO) and Vastus Lateralis (VL) muscles, which are especially pronounced during contraction. And PFP subjects exhibited significantly decreased muscle stiffness of VMO compared to asymptomatic limbs, with elevated muscle stiffness in the VL compared to asymptomatic limbs and healthy controls during the stretching. In terms of diagnostics, our study found that integrating VMO/VL muscle stiffness from both stretching and contraction positions holds significant potential for enhancing diagnostic accuracy in PFP.

## Supplementary Information

Supplementary Material 1.

## Data Availability

The datasets supporting the conclusions of this manuscript are fully available within the article and its supplementary materials. Additional raw data can be provided by the corresponding author upon reasonable request.

## Abbreviations

PFP: Patellofemoral pain
SWE: Shear Wave Elastography
sEMG: Surface Electromyography
VMO: Vastus Medialis Oblique
VL: Vastus Lateralis
RF: Rectus Femoris
PFJ: Patellofemoral Joint
US: Ultrasound
AKPS: Anterior Knee Pain Scale
KOOS-PF: Knee injury and Osteoarthritis Outcome Score - Physical Function Short Form
RMS: Root mean square
AUC: Area Under the Curve
